# 40LoVe and Samba Are Involved in *Xenopus* Neural Development and Functionally Distinct from hnRNP AB

**DOI:** 10.1371/journal.pone.0085026

**Published:** 2014-01-15

**Authors:** Maria Andreou, Chao Yun Irene Yan, Paris A. Skourides

**Affiliations:** 1 Department of Biological Sciences, University of Cyprus, Nicosia, Cyprus; 2 Department of Cell and Developmental Biology, Universidade de São Paulo, São Paulo, Brazil; School of Biomedical Sciences, The University of Queensland, Australia

## Abstract

Heterogeneous nuclear ribonucleoproteins (hnRNPs) comprise a large group of modular RNA-binding proteins classified according to their conserved domains. This modular nature, coupled with a large choice of alternative splice variants generates functional diversity. Here, we investigate the biological differences between 40LoVe, its splice variant Samba and its pseudoallele hnRNP AB in neural development. Loss of function experiments lead to defects in neural development with reduction of eye size, which stem primarily from increased apoptosis and reduced proliferation in neural tissues. Despite very high homology between 40LoVe/Samba and hnRNP AB, these proteins display major differences in localization, which appear to be in part responsible for functional differences. Specifically, we show that the 40Love/Samba carboxy-terminal domain (GRD) enables nucleocytoplasmic shuttling behavior. This domain is slightly different in hnRNP AB, leading to nuclear-restricted localization. Finally, we show that shuttling is required for 40LoVe/Samba function in neural development.

## Introduction

Heterogeneous nuclear ribonucleoproteins (hnRNPs) comprise a large group of RNA-binding proteins characterized by their modularity and biological versatility [1]. Most hnRNPs are composed of three domains with specific roles: a core RNA-binding domain (RBD or RRM-RNA recognition motif); RGG boxes that are composed of Arginine-Glycine-Glycine triplets interspersed with aromatic residues and a variable carboxy-terminal domain [1], [2]. The c-terminus is in some cases enriched with glycine, proline or acidic residues which depending on the specific function of the protein [2], [3]. Specifically, the glycine-rich domain (GRD) found at the c-terminus of a number of hnRNPs has been shown to be required for self-interaction and to be essential for the splicing activity of in the case of hnRNP A1 [4], to contain a non classical nuclear localization signal and promote nucleocytoplasmic shuttling and nuclear import in the case of hnRNP H/F [5]–[7]. Finally, several hnRNPs contain a third auxiliary domain that is variable [1] and in some hnRNPs this is a CARG-binding factor A domain (CBFNT), which has been shown interact with the promoter of immunoglobulin K [8].

Despite their structural similarities, hnRNPs participate in a diversity of cellular processes, including but not limited to alternative splicing, miRNA regulation, as well as mRNA compartmentalization and transport [1]. hnRNPs have also been shown to play multiple roles during embryonic development. For instance, Vg1-RBP/Vera and 40LoVe are essential for RNA compartmentization in the Xenopus oocyte [9]. In later stages Vg1-RBP is required for neural crest cell migration in the developing embryo [10]. Likewise, Samba is also expressed maternally and is later involved in the neural and neural crest development [11].

The presence of different hnRNPs, with similar structural features, in the same embryonic tissues, raises the intriguing possibility that they might play redundant roles in similar processes. Alternatively, similar hnRNPs might contribute to distinct biological processes, despite their high degree of homology. Thus, in this study, we investigate the biological functions of 40LoVe, its splice variant Samba and its pseudoallele hnRNP AB in amphibian neural development. We show that the subcellular localization and biological roles of 40LoVe and Samba are indistinguishable, but are clearly distinct from those of hnRNP AB. Finally, we show that these differences are due to slight differences in the GRD domain which confer different localization and ability for nucleocytoplasmic shuttling.

## Materials and Methods

### Cell culture and transfections

The *Xenopus* cell line XL177 [12] (kindly provided by Dr. Niovi Santama, University of Cyprus) was grown in L-15 medium Leibovitz plus 15% FBS and 100 mM L-Glutamine at RT. Transfections of XL177 cells were performed by electroporation according to the manufacturer's protocol (Invitrogen). Cells were plated on charged glass coverslips for all experiments.

### Embryos, microinjections and explants

*Xenopus laevis* embryos from induced spawning were staged according to Nieuwkoop and Faber (1967). Embryos were fertilized in vitro and dejellied using 1.8% L-cysteine, pH 7.8, then maintained in 0.1x Marc's Modified Ringer's (0.1xMMR). Microinjections were performed in 4% Ficoll in 0.3xMMR according to established protocols. Capped mRNAs were in vitro transcribed using mMessage machine (Ambion). The injections amounts per embryo were the following: GFP tagged 40LoVe, Samba and hnRNP AB and protein mutants 100 pg –200 pg, Rescue constructs of 40LoVe, Samba and hnRNP AB 80 pg. After the injections the embryos were cultured in 4% Ficoll in 0.33x MMR until stage 8 and then cultured in 0.1x MMR at room temperature.

### DNA constructs and morpholinos

All plasmids were constructed using standard molecular biology techniques and were sequenced. All primers and constructs used are listed in Table S1. The rescue constructs were generated by modifying the 5′ of the mRNAs, using synonym codons, so that the protein sequence was not affected. The GFP sequence was isolated from pEGFP-N1. Antisense morpholino oligonucleotides (MOs) were designed and ordered from Gene Tools, LLC. The sequences of Samba Morpholinos are MO1: TATACTGCTGCTCGGAGTCGGACAT and MO2: AATCGCCAAATTCCTCCAAGCGGAC. The sequence of the control Morpholino (CoMO) is: CCTCTTACCTCAGTTACAATTTATA.

### RT-PCR

cDNA was prepared via reverse transcription (SuperScriptIII First strand synthesis, Invitrogen) from RNA extracted from MO1 injected and control embryos. PCR was carried out using specific primer pairs as indicated in Table S1, for 40LoVe, Samba, hnRNP AB, actin, for the markers chordin and Sox2 and for the probes Sox10 and Ntub.

### Whole mount in situ hybridization

Whole-mount *in situ* hybridization of *Xenopus* embryos was performed as it has been described by Smith and Harland (1991) [13]. Probes used were: XrX1, Sox10, and Ntub. Bright field images were captured on a Zeiss LumarV12 fluorescent stereomicroscope.

### Whole mount TUNEL assay

TUNEL assay of Xenopus embryos was performed according to the Harland protocol (Conlon lab) available in Xenbase. Stored embryos were rehydrated and washed in 1xPBS and incubated for 1 hour at RT in TdT buffer (Invitrogen). Then, 150 U/ml TdT enzyme (Invitrogen) and 0.1 µl of DdUTP (Roche) per 100 µl buffer were added to the buffer solution and the embryos were incubated overnight at RT. The next day, embryos were washed 2x1 hour at 65°C in 1 mM EDTA/PBS and in 1xPBS 4x1 hour at RT, followed by 2–10 min washes in 1xMAB. Then they were blocked in 2%BMB blocking solution for 1 hour at RT and incubated in a 1/3000 dilution of anti-digoxigenin AP antibody in BMB block for 4 hours RT or overnight at 4°C. Antibody was washed away by 5x1 hour washes in MAB. Endogenous phosphatases were blocked by 2x10 min washes in alkaline phosphatase buffer and then NBT/BCIP (Roche) was added to the embryos. Chromogenic reaction was stopped by a quick wash in 1XMAB and then the embryos were fixed overnight in 1xMEMFA at RT. The next day embryos were imaged after clearing in two parts Benzyl Benzoate and one part Benzyl Alcohol after dehydration (Murray's Clearing Medium) (2∶1 BB: BA).

### Immunofluorescence

For whole mount immunofluorescence, embryos were fixed in 10% 10XMEMFA (0.1 mM MOPS pH 7.4, 2 mM EGTA, 1 mM MgSO_4_, 3.7% formaldehyde), 10% formaldehyde and 80% water for 2 hours at room temperature and the vitelline envelope was removed manually. Embryos were permeabilized overnight in 1XPBS, 0.5% Triton, 1% DMSO (Perm solution) and blocked for 2 hours in 10% Normal Goat serum in Perm solution. Embryos were then incubated with primary antibodies. The primary antibodies used were: 40LoVe (CvH7, C579 kindly provided by Dr. Iain W. Mattaj, EMBL, Heidelberg, Germany), GFP (Invitrogen), DYKDDDDK Epitope Tag Antibody (Novus), Acetylated Tubulin (Santa Cruz) and Histone H3 [pSer10] Antibody (Novus). The incubation was performed overnight at 4°C. Embryos were then washed four times in Perm solution for 20 min, incubated for 2 hours RT with secondary antibodies. The secondary antibodies used were: Cy3 (Jackson Immunoresearch) and Alexa-488 (Invitrogen). Then the embryos washed four times in Perm solution for 20 min. Clearing of embryos was performed by immersing the embryos in 2∶1 BB: BA.

### Western blot and densitometry analysis

Protein lysates were prepared by homogenizing explants or embryos in ice cold RIPA lysis buffer (50 mM Tris-HCl pH7.4, 150 mM NaCl, 2 mM EDTA, 1% NP-40, 0.1% SDS, 1% deoxycholate 24 mM) supplemented with protease inhibitors (1 mM PMSF, Protease cocktail, Sigma). Homogenates were cleared by centrifugation at 15000 g for 30 min at 4°C [60]. The lysates were loaded on 12% SDS-polyacrylamide gels with the Kaleidoskope ladder (Bio-Rad, USA). The proteins were transferred onto nitrocellulose membrane, blocked in 5% Skim Milk Powder (Sigma) in TBST (1X TBS & 0.1% Tween). The blotting was performed by incubation of the primary antibodies in Block Solution for 1 hour at RT. The primary antibodies used were: 40LoVe (CvH7, C579), β-tubulin (E7-c, Hybridoma Bank), DYKDDDDK Epitope Tag Antibody (Novus). The blots were washed 3x15 min in TBST. Visualization was performed using HRP-conjugated antibodies (1 hour incubation RT) (Santa Cruz Biotechnology anti-rabbit and anti-mouse, USA) and detected with LumiSensor (GeneScript) on UVP iBox. For loading control a tubulin mouse monoclonal antibody (β-tubulin, E7-c, Hybridoma Bank) was used in every blot. Densitometry analysis was carried out using the Vision Works LS Software.

### Imaging analysis

Embryos were observed either under a Zeiss Axio Imager Z1 microscope, using a Zeiss Axiocam MR3 and the Axiovision software 4,8. This software was used to measure the eye diameter in images of control, morphant and rescued embryos. All measurements were carried out in the anterior to posterior direction. Optical sectioning was achieved using a Zeiss Apotome structure illumination system. In addition, a Zeiss LumarV12 fluorescent stereomicroscope and a laser scanning confocal LSM710 microscope (Zeiss) were used, where indicated.

The generation of the intensity profiles and the data analysis of FRAP and FLIP experiments were performed with the ZEN2010 software. FRAP experiments were conducted using the LSM 710 confocal microscope and a Plan-Apochromat 63x/1.40 Oil DIC M27 objective lens (Zeiss). The 488 nm laser line was used for GFP excitation and emission was detected between 493–538 nm. Relative recovery rates were compared using half time for recovery of fluorescence towards the asymptote. The fluorescence recovery curve was fitted by single exponential function, given by: F(t)  =  A(1−e^−R^) + B; where F(t) is the intensity at time t; A and B are the amplitudes of the time-dependent and time-independent terms, respectively; τ is the lifetime of the exponential term (time constant) and the recovery rate is given by R = 1/τ. Immobile fractions were calculated by comparing the intensity ratio in the bleached area, just before bleaching and after recovery. For the FLIP experiments, photobleached regions consisted of a rectangle enclosing the selected region of the cell, which was repetitively photobleached during the experiment.

## Results

### Temporal and spatial expression of the Samba, 40LoVe and hnRNP AB

**Figure 1:**
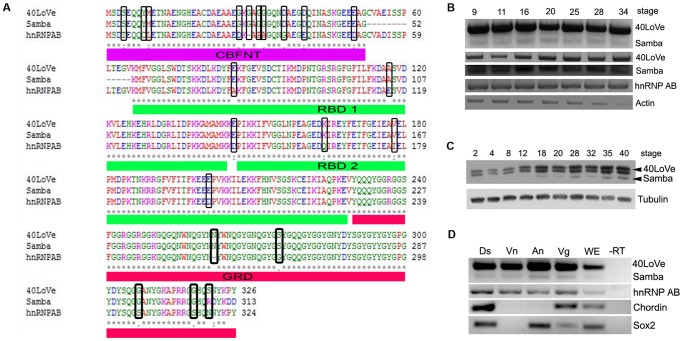
Alignments and Temporal Expression of 40LoVe, Samba and hnRNP AB. (A) Alignment of Samba, 40LoVe (GI: 240849376) and hnRNP AB (GI: 148224084) and domain specification. The N-terminus shows high homology with the CBFNT domain identified in the GARG-binding factor A protein [8]. There are two RNA binding domains, the RBD1 containing an RRM1 and the RBD2 containing an RRM2. At the C-terminus of the protein there is a Glycine Rich Domain (GRD). The comparison of hnRNP AB with 40LoVe shows that they have an overall 93% identity at the amino acid level with the differences spread throughout the sequence and indicated here inside the black boxes. (B) Characterization of temporal expression patterns of the three transcripts using RT-PCR. Primers that amplified nucleotides 1-350 (containing the 69 nucleotide deletion in Samba) were used to detect 40LoVe/Samba expression and primers spanning the 5′ UTR to the stop codon were used for RT-PCR detection of hnRNP AB. The first row shows the gel exposed in such a way that allows visualization of both 40LoVe and Samba. Two bands are visible: a strong high molecular weight band corresponding to 40LoVe and a weak lower molecular weight band corresponding to Samba. Rows two and three show 40LoVe and Samba exposed separately so as to clarity expression levels changes and the fourth row shows hnRNP AB levels. 40LoVe, Samba and hnRNP AB are expressed throughout development. Actin was used as a loading control. (C) Western Blot of half embryo equivalent indicates that the three proteins are expressed throughout development and confirms that the expression levels of 40LoVe are higher than those of Samba and that they share similar temporal regulation. Tubulin was used as a loading control. (D) RT-PCR from different regions dissected from a stage 10.5 embryo. 40LoVe/Samba are expressed in all regions of the embryo and hnRNP AB is expressed in a similar manner. The dorsal mesodermal marker Chordin and the neural marker Sox2 were used as controls. (Ds, dorsal region; Vn, Ventral region; An, Animal pole; Vg, Vegetal pole; WE, whole embryo; -RT, samples without reverse transcriptase)

*Xenopus laevis* genes frequently have pseudoalleles thought to have originated from hybridization between two different *Xenopus* species in the course of evolution [14]. Through an EST database search, we found that there are at least three very similar transcripts of Samba, a Xenopus hnRNP we recently characterized (Samba, 40LoVe and hnRNP AB) [11]. In agreement with this another group, has shown that 40LoVe displays three different isoforms during oogenesis that do not result from post-translational modifications [15]. Moreover, members of the hnRNP A/B-D subfamily frequently undergo alternative splicing to generate multiple isoforms [15], [16]. 40LoVe and Samba are identical both at the nucleotide as well as the amino acid level except for a 13 amino acid deletion at the N-terminus of Samba, between the CBFNT domain [8] and the RBD1 (Figure 1A). In addition, both the 3′ and 5′ UTR sequences of the two transcripts are identical, suggesting that these two proteins are splice variants. A comparison of hnRNP AB with 40LoVe shows that it has an overall 93% identity at the amino acid level with the differences concentrated at the amino and carboxy-termini (Figure 1A). However, although the 3′ and 5′ UTRs of hnRNP AB present significant homology to that of 40LoVe/Samba, they are not identical (data not shown). Together, these data suggest that hnRNP AB is a pseudoallele of 40LoVe/Samba.

To determine if these three transcripts display any differences with respect to their temporal expression, we carried out RT-PCRs. To distinguish between the Samba and 40LoVe transcripts, we specifically designed a set of primers corresponding to nucleotides 1-350 (the span which contains the 69 nucleotide deletion in Samba). RT-PCR amplification with this set of primers generates two bands as expected, a lower band that corresponds to Samba and a higher band that corresponds to 40LoVe (Figure 1B). As shown, 40LoVe appears to be the primary transcript of the gene giving a much stronger signal than Samba and both transcripts are present at all stages examined (Figure 1 B). Same holds true for the transcript of hnRNP AB. These results indicate that neither transcript presents significant changes in expression levels during development and that both 40LoVe and Samba are expressed throughout development albeit at different levels (Figure 1B). We went on to use a 40LoVe polyclonal antibody in order to examine the temporal expression at the protein level. As shown in Figure 1C the antibody produces three bands the lowest of which is at the predicted size for Samba, while the other two are at the predicted size for 40LoVe and hnRNP AB. The blot shows that in agreement with the RT-PCR results these proteins are expressed throughout development with protein levels gradually increasing and that the protein levels of Samba are much lower than those of 40LoVe.

Due to the very high homology of the three transcripts it was not possible to determine potential spatial differences of expression using *in situ* hybridization. For this reasons using the same primers, we compared the expression of the two transcripts in different regions of the embryo. 40LoVe, Samba and hnRNP AB are expressed in all regions of a stage 10,5 embryo as described before for Samba [11] (Figure 1D).Thus, it appears that 40LoVe and Samba are under a common spatiotemporal control, but with a clear bias towards generation of 40LoVe. When we examined hnRNP AB expression in the different regions of the embryo we concluded that its spatial and temporal expression dynamics are similar to those of 40LoVe and Samba (Figure 1B–D).

As stated above due to the high degree of homology, the spatial expression of the three transcripts could not be further differentiated via *in situ*. However, since both Samba and hnRNP AB have been reported to play a role in neural development, we performed functional assays focused on neural development [11], [17].

### Downregulation of 40LoVe/Samba causes cephalic and neuronal defects

**Figure 2:**
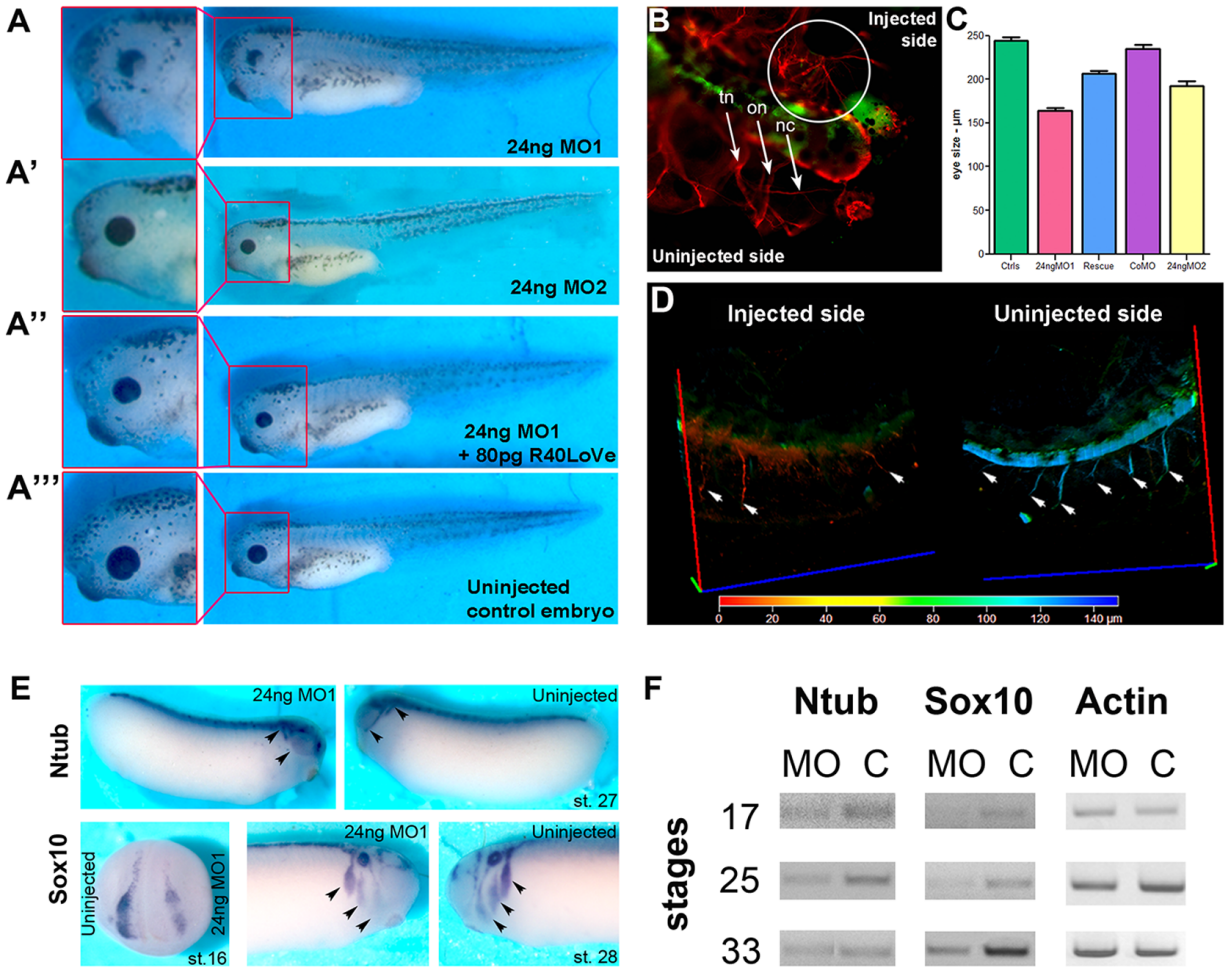
Downregulation of 40LoVe/Samba causes several head and neuronal defects. (A) 40LoVe/Samba MO1 morphants display generalized head defects with the most prominent being a reduced eye size, eye size, dorsal pigmentation, craniocephalic shape and overall cranial volume. (A’) MO2-injected embryo (24 ng) displayed a milder phenotype compared to MO1-injected embryos. (A’’) Co-injection of 80 pg of R40LoVe with 24 ng MO1 partially rescues the phenotype. (A’’’) Uninjected control embryo. Eye size was measured from embryos injected with MO1, MO2, control morpholino (CoMO), rescued (R2-R40Love co-injected with MO1 and R5-RhnRNPAB-GRD_40LoVe_ co-injected with MO1 as shown in table 1) and control embryos (n = 80–120 for each category). (B) Graph shows that in MO1-injected embryos the eyes were 40% smaller than controls and it was successfully rescued by injection of R40LoVe mRNA (Rescue 2 - eye size 90% of controls). hnRNPAB-GRD_40LoVe_ (Rescue 5) is also able to partially rescue the phenotype. (C) Optical sections from representative whole mount immunostained embryo for acetylated tubulin (red) to reveal neurons. The presence of using GFP (green) indicates the MO-injected side of the embryo (n = 20, three independent experiments). Compared to the uninjected side, the trigeminal nerve (tn), the ophthalmic nerve (on) and the nasocilliary nerves (nc) on the injected side of the embryo (marked with a white circle) are thinner and disorganized. The arrows show the properly formed neurons on the uninjected side of the tadpole. (D) Depth color coded (depth key at bottom of D) 3D reconstruction of optical sections of the trunk of a representative tadpole injected in the animal pole of one out two animal blastomeres with MO1 and then stained using an anti-acetylated tubulin antibody to reveal the axonal projections (n = 30, three independent experiments) 3D reconstruction is shown on the injected side (left) and was then rotated 180 degrees and shown on the uninjected side (right). Motor neuron projections rising from the spinal cord on the injected side of the embryo are absent or short in contrast to the un-injected side. (E) Whole mount *in situ* hybridization using N-tubulin (Ntub) and Sox10 show that the neural tissues are defined normally in MO1-injected embryos. However, tissues such as the cranial sensory ganglia and the branchial arches are misshapen and did not migrate normally (arrowheads) and expression levels Ntub and Sox 10 appear reduced in the injected side. (F) RT-PCR experiments confirm a reduction of neural marker expression in MO1 injected embryos. Actin was used as a loading control.

In order to address the role of 40LoVe/Samba and hnRNP AB in embryonic development, we generated a morpholino designed to block translation of all three transcripts (MO1). As shown MO1 downregulates both a surrogate 40LoVe/Samba and hnRNP AB and western blotting using the 40LoVe polyclonal antibody shows that all three bands recognized by this antibody are affected by the morpholino suggesting that MO1 blocks the translation of the endogenous proteins (Figure S1A, C). Immunofluorescence staining of MO1 injected tadpoles using the 40LoVe polyclonal antibody confirms downregulation in memGFP lineage traced cells that have received the morpholino compared to neighboring control cells (Figure S1D). Injections with MO1 led to mild head malformations and prominently reduced eyes (Figure 2A). Co-injection of MO1 with 80 pg of R40LoVe, which lacks the region recognized by MO1, led to an overall rescue of the phenotype (Figure 2A’’ and Table 1). Surprisingly co-injection of 80 pg of RhnRNP AB failed to rescue the head defects elicited by MO1 (Table 1) suggesting that the phenotype elicited is due to loss of 40LoVe and that despite the high homology, hnRNP AB and 40LoVe may be functionally distinct.

**Table 1:** Injected embryos with MO1, MO2, Control MO and rescue attempts with different constructs.

	Normal head structures (%)	Total number of embryos
24 ng MO1	4%	104 (n = 5)
24 ng MO2	30%	85 (n = 5)
24 ng MO1 + R1	36%	83 (n = 2)
24 ng MO1+ R2	50%	112 (n = 4)
24 ng MO1 + R3	5%	71 (n = 3)
24 ng MO1 + R4	4%	55 (n = 2)
24 ng MO1 + R5	42%	110 (n = 2)
CoMO	83%	84 (n = 2)
Uninjected Controls	90%	66 (n = 4)

R1  =  60 pg RSamba + 60 pg R40LoVe.

R2  =  80 pg R40LoVe.

R3  =  80 pg RhnRNPAB.

R4  =  80 pg R40LoVe-GRD_AB._

R5  =  80 pg RhnRNPAB-GRD_40LoVe._

Head structures were scored according to eye size, dorsal pigmentation, craniocephalic shape and overall volume. The phenotype was rescued in 50% of the embryos injected with 80 pg of R40LoVe and 42% of the embryos injected with 80 pg of RhnRNPAB-GRD_40LoVe_. The transcripts whose products are exclusively nuclear (e.g. RhnRNP AB and R40LoVeGRD_AB_) cannot rescue the phenotype. The experiments were performed n times for each case as indicated and the numbers on the table are the mean for each case.

Detailed analysis of cephalic innervations in morphants showed that the cranial neurons in the MO1-injected side were not properly formed. The staining pattern for beta-tubulin in the morphant side was disorganized and thinner compared to those in the uninjected side (Figure 2C). Furthermore, motor neurons rising from the spinal cord on the injected side of the embryo were absent in contrast to the uninjected side (Figure 2D). We also noticed that crest derivatives such as the brachial arches did not migrate properly in morphants (Figure 2E). To address the possibility that the observed phenotypes were caused by alterations in neuronal differentiation, we carried out WISH using neural markers. Although all the markers examined were expressed, their expression domain was reduced in the MO injected side of the embryo (Figure 2E). RT-PCR experiments confirmed a reduction in neural marker expression in MO injected embryos (Figure 2F). These data suggest that 40LoVe/Samba is not required for neural specification, but may be required for maintenance or survival of neuronal tissues in the embryo.

**Figure 3:**
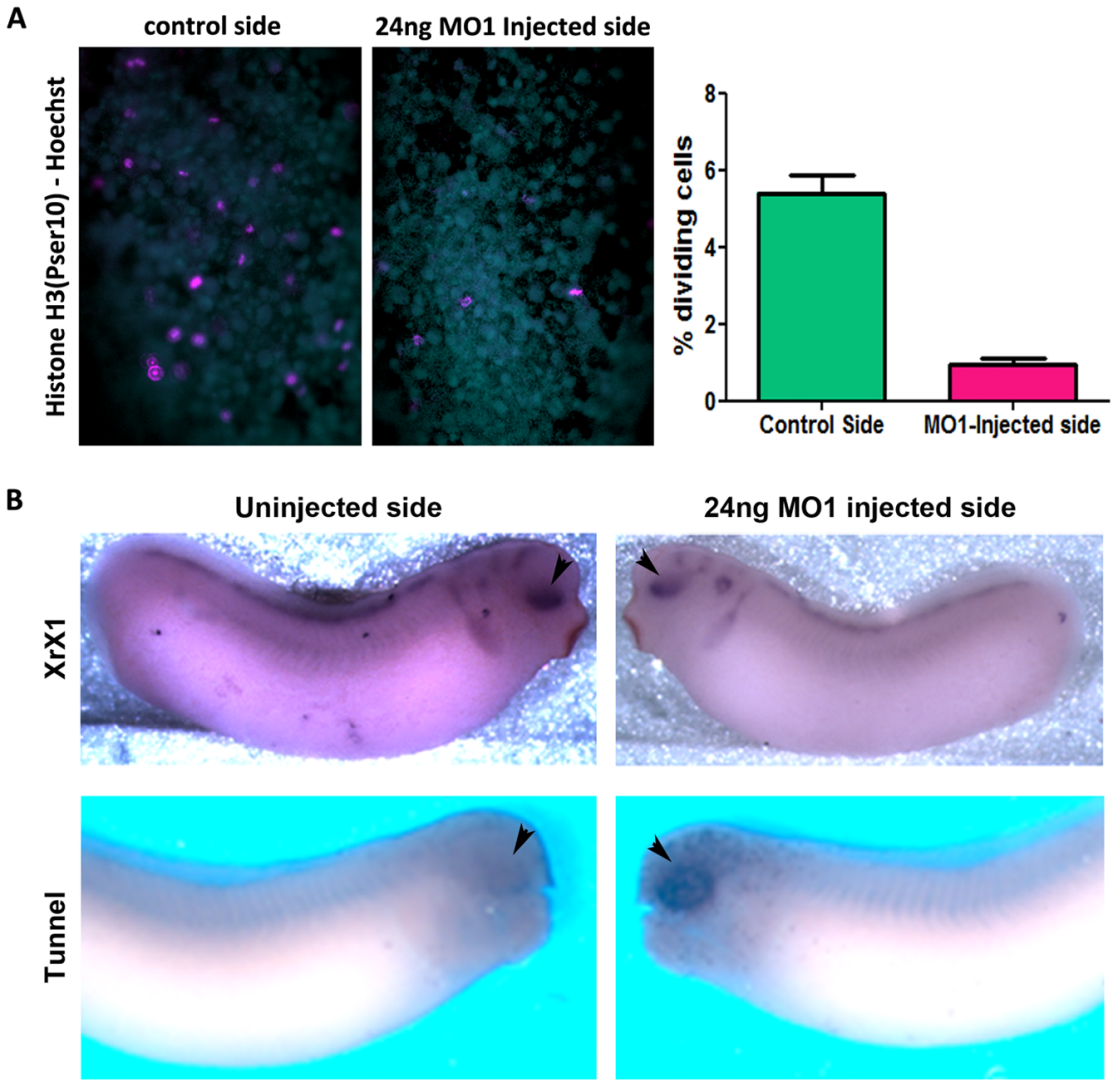
40LoVe/Samba are required for neuronal cell survival. (A) Hoechst (cyan) and Histone H3[p Ser10] (magenta) staining reveals mitotic cells of a representative embryo injected with MO1 in one out of two animal blastomeres at the two cell stage. The images show that fewer dividing cells are present in the MO1 injected side of the embryo compared to the uninjected side of the same embryo. The graph shows quantification from 15 embryos from two independent experiments. (B) Whole mount *in situ* hybridization using the specific eye marker XrX1 shows reduced expression in the eye on the injected side compared to the control side. TUNEL staining reveals increased apoptosis in the eye and other head structures in MO1-injected side of the embryo compared to the uninjected side of the same embryo.

We went on to examine the rate of cell division in injected embryos using a mitotic cell marker, Histone H3 [p Ser10] and noted a reduction in the number of dividing cells in the MO injected side of the embryo (Figure 3A). TUNEL staining also revealed increased apoptosis in the eye and other head structures in MO-injected embryos (Figure 3B). These results suggest that the reduced eye size and the decreased neural marker expression in morphants is due to the selective loss of neural tissues through apoptosis and suggest that 40LoVe/Samba, but not hnRNP AB, are required for neuronal cell survival. In order to confirm that the observed neuronal defects are in fact due to loss of 40LoVe/Samba and are unrelated to the concomitant downregulation of hnRNP AB, we went on to take advantage of differences in the UTRs and generate a 40LoVe/Samba specific morpholino (MO2). As shown while MO1 downregulates both a surrogate 40LoVe/Samba and hnRNP AB, MO2 fails to downregulate hnRNP AB but downregulates 40LoVe/Samba albeit with a lower efficiency (Figure S1B). Despite the inability of MO2 to downregulate hnRNP AB, it induces an identical phenotype to MO1 confirming that neuronal defects elicited by MO1 are in fact due to loss of 40LoVe/Samba (Figure 2A’).

### Protein localization at the cellular level

**Figure 4:**
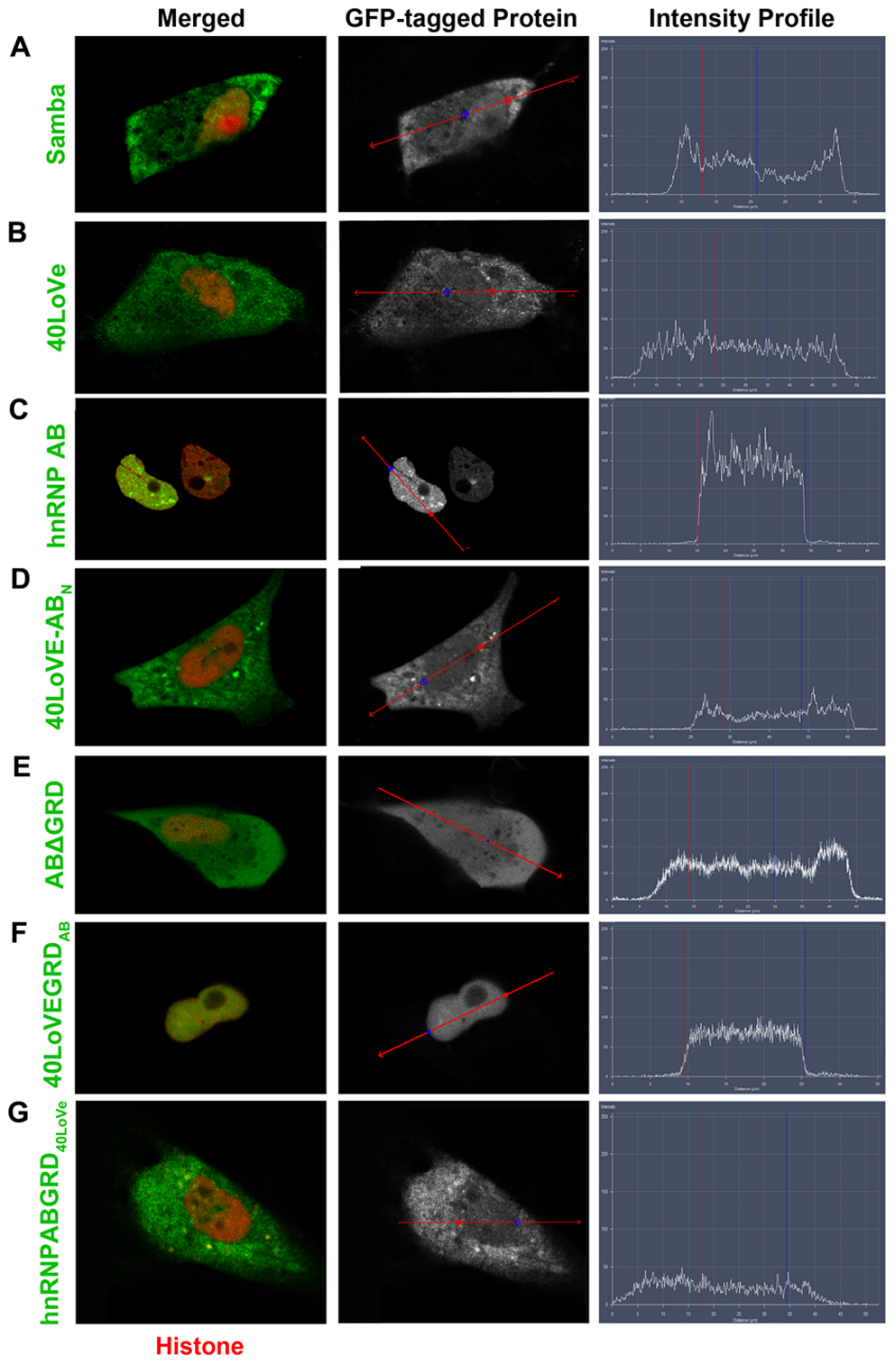
40LoVe/Samba, hnRNP AB, deletion mutant and fusion construct localization in XL177 cells. In each figure cells were co-electroporated with a GFP-tagged version of the protein indicated on the left (green) and Histone-RFP to label the nucleus (red). The first column shows the merged image of both the indicated construct (green) and histone (red), while the second column shows the construct localization alone. The third column shows the intensity profile for each cell along the red line shown in the second column. Red and blue lines represent the boundaries of the nucleus of each cell. (A–B) Samba and 40LoVe are localized both in the nucleus and the cytosol. (C) hnRNP AB is exclusively nuclear. (D) 40LoVe-AB_N_ localizes like 40LoVe, suggesting that the N-terminus of the protein is not responsible for nuclear retention of hnRNP AB. (E) ABΔGRD loses the strictly nuclear localization of hnRNP AB, indicating that the GRD domain is necessary for the exclusively nuclear localization of hnRNP AB. (F) 40LoVeGRD_AB_ localizes exactly like hnRNP AB, showing that the GRD domain of hnRNP AB is sufficient for nuclear retention. (G) hnRNP AB-GRD_40LoVe_ localizes like 40LoVe, confirming that the hnRNP AB GRD is necessary for nuclear retention of hnRNP AB and that that the few amino acid differences between the GRD domains of the two proteins are responsible for their differences in localization.

The above results suggested that 40LoVe and hnRNP AB are functionally distinct a surprising finding given their very high homology. To determine if the observed functional differences are due to differences in subcellular localization, we compared the distribution of GFP-tagged Samba, 40LoVe and hnRNP AB. As shown Samba is found at the plasma membrane, the nucleus, and the cytoplasm (Figure 4A) and 40LoVe displayed a similar overall localization (Figure 4B). Surprisingly, hnRNP AB was exclusively localized in the nucleus and no membrane or cytoplasmic signal was detected in the majority of cells (Figure 4C). Taken together, these data show that the three proteins exhibit major differences in localization which may underlie the functional differentiation observed in the loss of function experiments.

### Mapping the domains responsible for the protein localization

Given the fact that the localization of 40LoVe/Samba is strikingly different from that of hnRNP AB, we generated mutants and chimeras to identify the domain responsible for these differences (Figure S2). Since most of the differences (10/21 amino acids) between 40LoVe/Samba and hnRNP AB are found at the N-terminus (CBFNT domain), we exchanged the N-terminus of 40LoVe/Samba with the N-terminus of hnRNP AB. The chimera (40LoVeAB_N_) localizes like 40LoVe displaying membrane cytosolic and nuclear staining (Figure 4D), suggesting that the region responsible for the exclusively nuclear localization (of hnRNP AB) is not the N-terminus of the protein.

Since the GRD domains of several hnRNPs have been shown to be important for their localization [3], [7], [18], we deleted the GRD domain of hnRNP AB. The hnRNP AB mutant (ABΔGRD), unlike the full length protein, is not exclusively nuclear (Figure 4E) suggesting that the GRD domain is necessary for the accumulation of hnRNP AB into the nucleus. The above results also suggest that the few amino acid differences between the GRD domains of 40LoVe and hnRNP AB (5 amino acids shown in Figure 1A) are responsible for the dramatic difference in localization. To examine this possibility further we exchanged the GRD of 40LoVe with the GRD of hnRNP AB (40LoVe-GRD_AB_). The resulting chimera behaved according to the GRD donor. 40LoVe-GRD_AB_ was excluded from the cytoplasm and localized exclusively in the nucleus (Figure 4F). These results show that the GRD domain is both necessary and sufficient for the accumulation of hnRNP AB in the nucleus. Examination of the GRD of hnRNP AB for previously identified NLS sequences failed to reveal any classical nuclear localization signal. However these results suggest that such a signal does in fact reside within the GRD domain of hnRNP AB and further mutational analysis will be required to determine the exact residues within the domain responsible for this activity.

**Figure 5:**
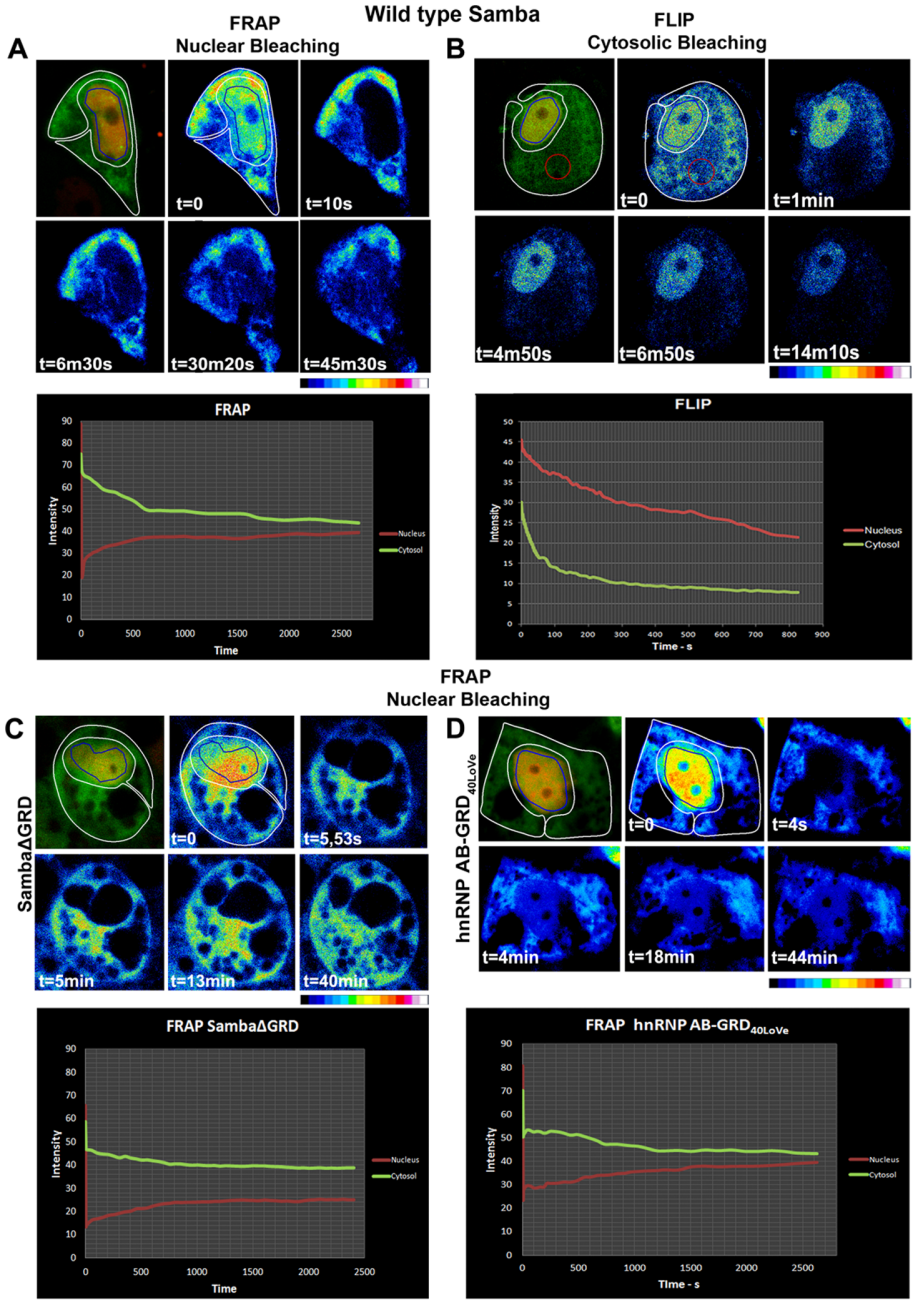
40LoVe/Samba shuttles from the nucleus to the cytosol and the GRD domain is both necessary and sufficient for this process. (A) Intensity coded still images (intensity scale is shown below the set of images) from a time lapse recording of a *FRAP* experiment in a cell co-expressing GFP-Samba and Histone RFP. The area outlined in blue (the nucleus) was bleached and the GFP signal intensity was monitored over time both in the nucleus (blue outline) and the cytosol (white outline). The graph shows the fluorescence intensity in the nucleus and the cytosol. The intensity difference between the two compartments is nearly extinguished after about 40 minutes. After 40 minutes the nuclear signal has recovered while the cytsosolic signal has decreased showing that cytosolic protein moved into the nucleus and suggesting that bleached molecules are exiting the nucleus and moving into the cytosol. (B) Intensity coded still images (intensity scale is shown below the set of images) from a time lapse recording of a *FLIP* experiment. When the cytosol (white outline) is repeatedly bleached (red circle), signal intensity in the nucleus (outline) diminishes gradually confirming that molecules in the nucleus are moving into the cytosol. The graph shows the fluorescence intensity in the nucleus (blue outline) and the cytosol (white outline). (C) Intensity coded still images (intensity scale is shown below the set of images) from a time lapse recording of a *FRAP* experiment from a GFP-SambaΔGRD expressing cell. Unlike full length Samba the signal intensity in the nucleus fails to recover while the signal in the cytosol also fails to decrease suggesting that SambaΔGRD does not shuttle from the cytoplasm to the nucleus. The graph shows the fluorescence intensity in the nucleus (outlined blue) and cytosol (outlined white) over time. The intensity difference between the two compartments is reduced but fails to be extinguished even after about 40 minutes. (D) Intensity-coded still images (intensity scale is shown below the set of images) from a time lapse recording of a *FRAP* experiment from an hnRNPAB-GRD_40LoVe_ expressing cell. Nuclear signal recovers while cytosolic signal decreases over time in a similar fashion to what is observed with GFP-Samba suggesting that the GRD domain of 40LoVe is sufficient to confer shuttling from the nucleus to the cytoplasm. The graph was generated from measurements in the cytosol (outlined white) circle and the nucleus (outlined blue). The intensity difference between the two compartments is nearly extinguished after about 40 minutes showing similar dynamics to GFP-Samba (A).

The localization of 40LoVe in the cytosol and the nucleus raised the possibility that the protein shuttles between the two compartments. In order to examine this we used GFP fusions of both 40LoVe and Samba. Cells expressing GFP tagged full length 40LoVe and Samba presented GFP-fluorescence in both the nucleus and cytoplasm as described above. Using fluorescence recovery after photobleaching experiments we went on to examine the possibility of Nucleocytoplasmic shuttling. When the nuclear fraction of GFP-Samba was bleached nuclear fluorescence recovered in less than an hour, indicating that GFP-Samba shuttled from the cytoplasm to the nucleus during this period (FRAP; Figure 5A). Conversely, the nuclear signal gradually decreased when the cytosolic fraction of the protein was bleached in fluorescence loss in photobleaching experiments (FLIP; Figure 5B), suggesting that 40LoVe/Samba egressed from the nucleus to the cytoplasm. Overall these data show that 40LoVe/Samba undergo nucleocytoplasmic shuttling. Given the fact that nuclear retention of hnRNP AB is dependent on its GRD domain we went on to examine if shuttling of 40LoVe/Samba required the GRD domain. Removal of 40LoVe/Samba GRD (SambaΔGRD) completely blocked nucleocytoplasmic shuttling (FRAP; Figure 5C) suggesting that the GRD domain is necessary for 40LoVe/Samba shuttling. To determine if the 40LoVe/Samba GRD is also sufficient we generated an hnRNP AB in which the GRD domain was replaced with the 40LoVe/Samba GRD (hnRNP AB-GRD_40LoVe_; Figure 4G). This chimeric protein behaved like 40LoVe/Samba in FRAP experiments showing that the 40LoVe/Samba is both necessary and sufficient for nucleocytoplasmic shuttling (FRAP; Figure 5D).

Overall, the above results show that hnRNP AB and 40LoVe/Samba display major differences in localization which are a result of few amino acid differences between their respective GRD domains. While hnRNP AB is exclusively nuclear and its GRD domain is both necessary and sufficient to confer that localization 40LoVe/Samba are found both in the cytosol and the nucleus shuttling between the two compartments and their GRD domain is necessary for shuttling. These differences are likely the basis for the distinct function of the proteins in neural development in which case one would predict that nucleocytosplamic shuttling of 40LoVe/Samba must be functionally significant. Since hnRNP AB fails to rescue the morphants despite its very high homology to 40LoVe, we postulated that this maybe due to its strictly nuclear localization. To examine this possibility we took advantage of the 40LoVeGRD_AB_ chimera. Co-injection of the 40LoVeGRD_AB_ mRNA with MO1 failed to rescue the small eye phenotype elicited by the MO (Table 1). On the other hand co-injection of hnRNP AB-GRD_40LoVe_ with MO1 does rescue the small eye phenotype, albeit with lower efficiency than expression of 40LoVe, suggesting that the cytoplasmic localization and shuttling are at least partly responsible for the differences in function (Figure 2B, Table 1).

## Discussion

hnRNPs are defined by common motifs and the regions of sequence divergence amongst phylogenetically close variants are concentrated in the terminal-regions of the proteins [1]. Here, we report functional differences between two closely related hnRNPs which correlated with their subcellular localization. In turn, the latter is determined by point variations in the GRD domain.

40LoVe presents at least four different splice variants that result in protein product differences [15]. Amongst these, 40LoVe and its splice variant Samba differ in the presence of an insert between the CBFNT and RBD domain. 40LoVe/Samba is also closely related to hnRNP AB with differences at a total of 21 residues; 10 distributed in the amino-terminal CBFNT domain, 5 in the GRD domain and 6 in the central RBDs (RNA-binding domain). The 4.6% sequence difference observed between 40LoVe/Samba and hnRNP AB GRD domains was sufficient to produce a dramatic localization change and as a result make the two proteins functionally distinct.

Firstly, reduction in 40LoVe/Samba but not hnRNP AB levels resulted in a neural phenotype. The more striking features of the morphants were reduction in eye size and axonal growth. Overexpression of Samba gives a similar eye phenotype which was attributed to interference with neural crest migration [11]. The size of the migrating crest chains were also reduced in the morphants shown here, raising the intriguing possibility that an optimal level of 40LoVe/Samba expression is required for proper neural crest migration. Indeed, RNA binding proteins participate actively in the establishment of cell-substrate adhesion centers [19]–[22]. Both decrease or increase in hnRNP expression levels or activity inhibit cell spreading and migration [19]–[21]. Together, these data suggest that cell spreading depends on the proper balance of hnRNPs levels in the cytoplasm during spreading or migration.

The participation of hnRNPs in adhesive centers in the cytoplasm could also explain the second major difference that we detected between 40LoVe/Samba and hnRNP AB. In our experimental paradigm, hnRNP AB was strictly localized in the nucleus, while 40LoVe/Samba localized both in the nucleus and the cytosol and trafficked between the compartments. The nucleocytoplasmic shuttling and/or cytosolic localization was essential for the rescue of the morphants, as the 40LoVe-GRD_AB_ mutant that is restricted to the nucleus cannot rescue the morphant phenotype.

The 40LoVe/Samba nucleocytoplasmic shuttling behavior and nuclear retention of hnRNP A/B are conferred solely by their respective Glycine Rich Domains. In support of this, some hnRNPs have been reported to contain unconventional nuclear localization signals and shuttling sequences in their GRD domains [5], [23], [24]. A search for canonical NLS and NES signals in the 40LoVe and hnRNP AB GRD domains failed to detect any such sequences. However alignments of several characterized non canonical NLS and shuttling sequences revealed that 40LoVe and hnRNP AB have a sequence that is very similar to the DNS signal identified in hnRNP D [25]. This 19 amino acid sequence in 40LoVe and hnRNP AB presents an 80% sequence identity to DNS. Two interesting features arise from the alignment of the 40LoVe, hnRNP AB and hnRNP D C-terminal domains that might explain their differences in subcellular distribution (Figure S3). Firstly, all three proteins present a KPY motif that was identified as essential for nuclear import/retention of hnRNP D [25]. A second feature worthy of mention is the variation of 14^th^ residue in the hnRNP D DNS domain. This residue corresponds to Asparagine_350_ in hnRNP D and Asparagine_319_ in hnRNP AB. However, in 40LoVe and Samba, the corresponding residue is switched to Serine. hnRNP D localizes like hnRNP AB (strictly nuclear) unlike 40LoVe and Samba suggesting that this particular Asparagine residue is likely responsible for the differences in localization between 40LoVe/Samba and hnRNP AB. None of the other residue differences, between the hnRNP D DNS and hnRNP AB or 40LoVe/Samba, display the same correlation. This issue however can be further clarified with point mutations in future work. An additional point of interest is that while hnRNP D is strictly nuclear like hnRNP AB, use of heterokaryons showed that hnRNP D does shuttle between the nucleus and the cytosol [25]. This suggests that hnRNP AB may also shuttle and that the Asparagine to Serine change between 40LoVe and hnRNP AB, generates a weaker NLS that allows a large fraction of 40LoVe to be retained in the cytosol and that this cytosolic localization is essential for its function rather than shuttling per se. In addition the GRD domain sequence could also direct hnRNP localization indirectly, by determining the identity of their nucleic acid targets or protein partners [1].

Nevertheless, our results show that the few amino acid differences between 40LoVe/Samba and hnRNP AB GRD domains are sufficient to grant nuclear retention to the latter and this retention is at least in part responsible for the failure of hnRNP AB to rescue the neuronal phenotypes observed in morphants, since hnRNP AB-GRD_40LoVe_ is able to partially rescue the elicited small eye phenotype. More importantly, it also brings to attention that slight differences in the sequences of closely related hnRNPs are sufficient to generate different subcellular localization and, as a consequence, influence their biological functions.

## Supporting Information

Figure S1 **Morpholino downregulation of 40LoVe/Samba.** (A) Western Blot of half embryo equivalent injected with 60 pg of surrogate Samba-flag or hnRNP AB alone or co-injected with 8 ng, 16 ng and 24 ng MO1 as indicated. MO1 effectively downregulates both Samba/40LoVe and hnRNP AB. (B) Western Blot of half embryo equivalent injected with 60 pg of surrogate hnRNP AB or 40LoVe alone or co-injected with 8 ng, 16 ng and 24 ng MO1 as indicated. MO2 fails to downregulate hnRNP AB but downregulates 40LoVe/Samba. (C) Western Blot of half embryo equivalent injected with 12 ng, 24 ng and 30 ng MO1 shows that MO1 can effectively downregulate endogenous 40LoVe. Tubulin was used as a loading control. (D) Immunofluorescence experiments using the 40LoVe antibody confirm that MO1 down-regulates the endogenous protein. mGFP was used as a linage tracer of MO1 injected cells.
(TIF)

Figure S2 **Mutants generated for the determination of the protein domains responsible for the differences in localization between the three proteins.** 40LoVe-AB_N_ was constructed using the N-terminus of hnRNP AB form start to nucleotide 270/amino acid 90 and the rest of 40LoVe protein from nucleotide 273/amino acid 91 to the stop codon. ABΔGRD is hnRNP AB with a deleted c-terminus form nucleotide 630/amino acid 210 with an added stop codon. 40LoVeGRD_AB_ was constructed with 40LoVe from start codon to nucleotide 666/amino acid 222 fused with hnRNP AB from nucleotide 633/amino acid 221 to stop codon. hnRNP AB-GRD40LoVe was constructed with hnRNP AB from start codon to nucleotide 630/amino acid 210 fused with 40LoVe from amino acid 690/amino acid 230 to stop codon. All constructs are fused with a FLAG-tag at the C-terminus.
(TIF)

Figure S3 **Alignment of the GRD domains of 40LoVe/Samba, hnRNP AB and the human hnRNP D (GI: 51477711).** DNS is the 19 amino acid sequence highlighted in turquoise that has been shown to be responsible for shuttling in hnRNP D. Two out of the three differences in the GRD domain between 40LoVe/Samba and hnRNP AB are located in this 19 amino acid sequence. The yellow highlighted amino acid is the one likely responsible for differences in localization between 40LoVe/Samba and hnRNP AB. This amino acid is an Asparagine in hnRNP D and hnRNP AB, which both are strictly nuclear, but it's a Serine in 40LoVe/Samba which show both nuclear and cytosolic localization.
(TIF)

Table S1 Constructs and primers used for each construct generation and the RT-PCRs.
(DOCX)

## References

[1] Han SP, Tang YH, Smith R (2010) Functional diversity of the hnRNPs: past, present and perspectives. Biochem J 430: 379–392. 20795951 10.1042/BJ20100396

[2] Swanson MS, Nakagawa TY, LeVan K, Dreyfuss G (1987) Primary structure of human nuclear ribonucleoprotein particle C proteins: conservation of sequence and domain structures in heterogeneous nuclear RNA, mRNA, and pre-rRNA-binding proteins. Mol Cell Biol 7: 1731–1739. 3110598 10.1128/mcb.7.5.1731-1739.1987 PMC365274

[3] Cartegni L, Maconi M, Morandi E, Cobianchi F, Riva S, et al (1996) hnRNP A1 selectively interacts through its Gly-rich domain with different RNA-binding proteins. J Mol Biol 259: 337–348. 8676373 10.1006/jmbi.1996.0324

[4] Mayeda A, Munroe SH, Caceres JF, Krainer AR (1994) Function of conserved domains of hnRNP A1 and other hnRNP A/B proteins. EMBO J 13: 5483–5495. 7957114 10.1002/j.1460-2075.1994.tb06883.x PMC395506

[5] Van Dusen CM, Yee L, McNally LM, McNally MT (2010) A glycine-rich domain of hnRNP H/F promotes nucleocytoplasmic shuttling and nuclear import through an interaction with transportin 1. Mol Cell Biol 30: 2552–2562. 20308327 10.1128/MCB.00230-09 PMC2863714

[6] McNally LM, Yee L, McNally MT (2006) Heterogeneous nuclear ribonucleoprotein H is required for optimal U11 small nuclear ribonucleoprotein binding to a retroviral RNA-processing control element: implications for U12-dependent RNA splicing. J Biol Chem 281: 2478–2488. 16308319 10.1074/jbc.M511215200

[7] Kiledjian M, Dreyfuss G (1992) Primary structure and binding activity of the hnRNP U protein: binding RNA through RGG box. EMBO J 11: 2655–2664. 1628625 10.1002/j.1460-2075.1992.tb05331.x PMC556741

[8] Bemark M, Olsson H, Heinegard D, Leanderson T (1998) Purification and characterization of a protein binding to the SP6 kappa promoter. A potential role for CArG-box binding factor-A in kappa transcription. J Biol Chem 273: 18881–18890. 9668064 10.1074/jbc.273.30.18881

[9] Czaplinski K, Mattaj IW (2006) 40LoVe interacts with Vg1RBP/Vera and hnRNP I in binding the Vg1-localization element. RNA 12: 213–222. 16373488 10.1261/rna.2820106 PMC1370901

[10] Yaniv K, Fainsod A, Kalcheim C, Yisraeli JK (2003) The RNA-binding protein Vg1 RBP is required for cell migration during early neural development. Development 130: 5649–5661. 14522877 10.1242/dev.00810

[11] Yan CY, Skourides P, Chang C, Brivanlou A (2009) Samba, a Xenopus hnRNP expressed in neural and neural crest tissues. Dev Dyn 238: 204–209. 19097051 10.1002/dvdy.21826

[12] Miller L, Daniel JC (1977) Comparison of in vivo and in vitro ribosomal RNA synthesis in nucleolar mutants of Xenopus laevis. In Vitro 13: 557–563. 562840 10.1007/BF02627851

[13] Smith WC, Harland RM (1991) Injected Xwnt-8 RNA acts early in Xenopus embryos to promote formation of a vegetal dorsalizing center. Cell 67: 753–765. 1657405 10.1016/0092-8674(91)90070-f

[14] Kobel HR, Pasquier LD (1986) Genetics of polyploid Xenopus: Trends Genet

[15] Kroll TT, Swenson LB, Hartland EI, Snedden DD, Goodson HV, et al (2009) Interactions of 40LoVe within the ribonucleoprotein complex that forms on the localization element of Xenopus Vg1 mRNA. Mech Dev 126: 523–538. 19345262 10.1016/j.mod.2009.03.007

[16] Norvell A, Kelley RL, Wehr K, Schupbach T (1999) Specific isoforms of squid, a Drosophila hnRNP, perform distinct roles in Gurken localization during oogenesis. Genes Dev 13: 864–876. 10197986 10.1101/gad.13.7.864 PMC316593

[17] Sinnamon JR, Waddell CB, Nik S, Chen EI, Czaplinski K (2012) Hnrpab regulates neural development and neuron cell survival after glutamate stimulation. RNA 18: 704–719. 22332140 10.1261/rna.030742.111 PMC3312558

[18] Schaeffer C, Bardoni B, Mandel JL, Ehresmann B, Ehresmann C, et al (2001) The fragile X mental retardation protein binds specifically to its mRNA via a purine quartet motif. EMBO J 20: 4803–4813. 11532944 10.1093/emboj/20.17.4803 PMC125594

[19] de Hoog CL, Foster LJ, Mann M (2004) RNA and RNA binding proteins participate in early stages of cell spreading through spreading initiation centers. Cell 117: 649–662. 15163412 10.1016/s0092-8674(04)00456-8

[20] Yoo Y, Wu X, Egile C, Li R, Guan JL (2006) Interaction of N-WASP with hnRNPK and its role in filopodia formation and cell spreading. J Biol Chem 281: 15352–15360. 16574661 10.1074/jbc.M511825200

[21] Vikesaa J, Hansen TV, Jonson L, Borup R, Wewer UM, et al (2006) RNA-binding IMPs promote cell adhesion and invadopodia formation. EMBO J 25: 1456–1468. 16541107 10.1038/sj.emboj.7601039 PMC1440323

[22] Katz ZB, Wells AL, Park HY, Wu B, Shenoy SM, et al (2012) beta-Actin mRNA compartmentalization enhances focal adhesion stability and directs cell migration. Genes Dev 26: 1885–1890. 22948660 10.1101/gad.190413.112 PMC3435492

[23] Siomi H, Dreyfuss G (1995) A nuclear localization domain in the hnRNP A1 protein. J Cell Biol 129: 551–560. 7730395 10.1083/jcb.129.3.551 PMC2120450

[24] Weighardt F, Biamonti G, Riva S (1995) Nucleo-cytoplasmic distribution of human hnRNP proteins: a search for the targeting domains in hnRNP A1. J Cell Sci 108 (Pt 2): 545–555. 7769000 10.1242/jcs.108.2.545

[25] Suzuki M, Iijima M, Nishimura A, Tomozoe Y, Kamei D, et al (2005) Two separate regions essential for nuclear import of the hnRNP D nucleocytoplasmic shuttling sequence. FEBS J 272: 3975–3987. 16045768 10.1111/j.1742-4658.2005.04820.x

