# Supplementary figures and images for: 40LoVe and Samba Are Involved in Xenopus Neural Development and Functionally Distinct from hnRNP AB
Source: PLoS One. 2014 Jan 15;9(1):e85026. doi: 10.1371/journal.pone.0085026 (PMC3893134; doi:10.1371/journal.pone.0085026)

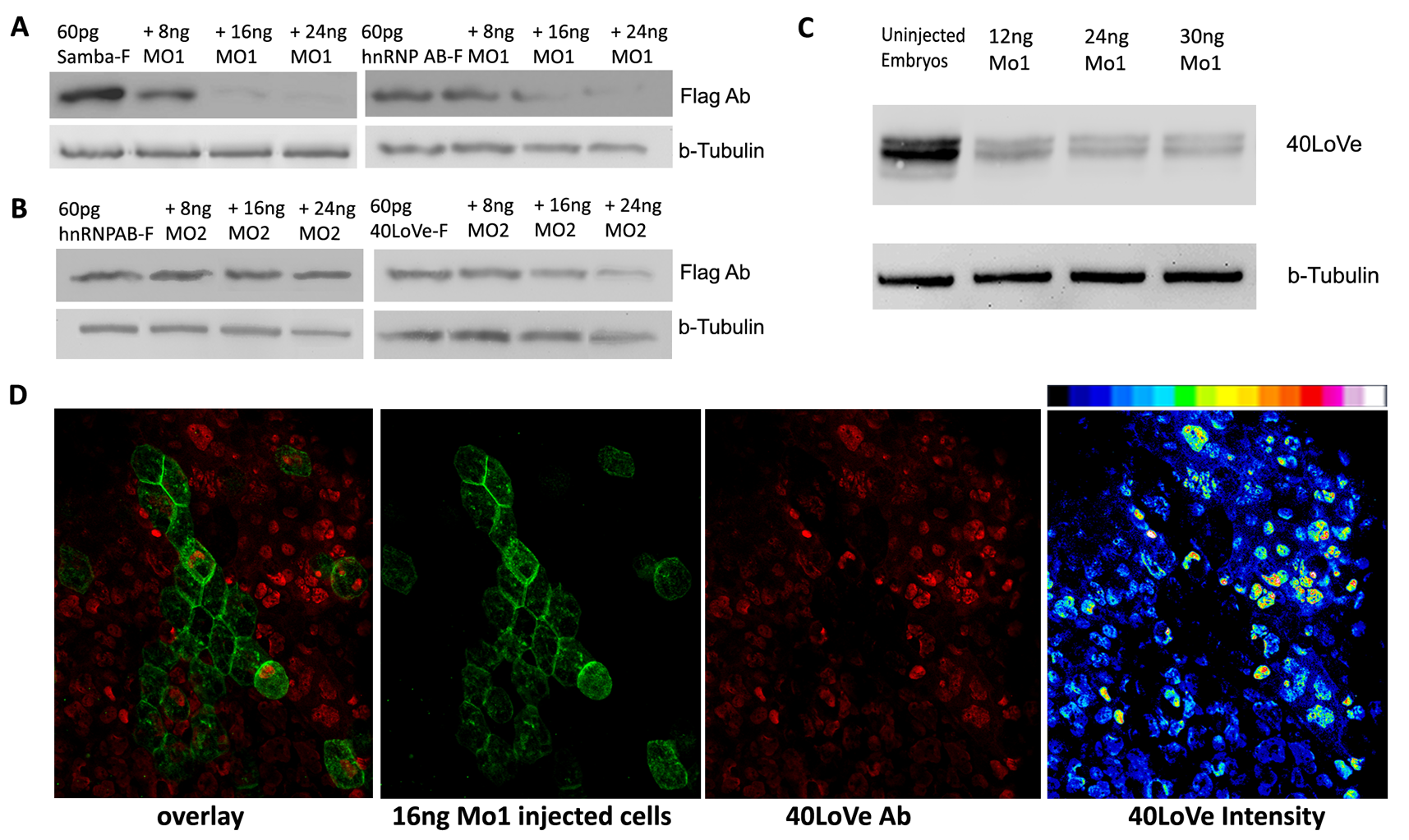

Supplement: Figure S1 — Morpholino downregulation of 40LoVe/Samba. (A) Western Blot of half embryo equivalent injected with 60 pg of surrogate Samba-flag or hnRNP AB alone or co-injected with 8 ng, 16 ng and 24 ng MO1 as indicated. MO1 effectively downregulates both Samba/40LoVe and hnRNP AB. (B) Western Blot of half embryo equivalent injected with 60 pg of surrogate hnRNP AB or 40LoVe alone or co-injected with 8 ng, 16 ng and 24 ng MO1 as indicated. MO2 fails to downregulate hnRNP AB but downregulates 40LoVe/Samba. (C) Western Blot of half embryo equivalent injected with 12 ng, 24 ng and 30 ng MO1 shows that MO1 can effectively downregulate endogenous 40LoVe. Tubulin was used as a loading control. (D) Immunofluorescence experiments using the 40LoVe antibody confirm that MO1 down-regulates the endogenous protein. mGFP was used as a linage tracer of MO1 injected cells. (TIF) [file pone.0085026.s001.tif]

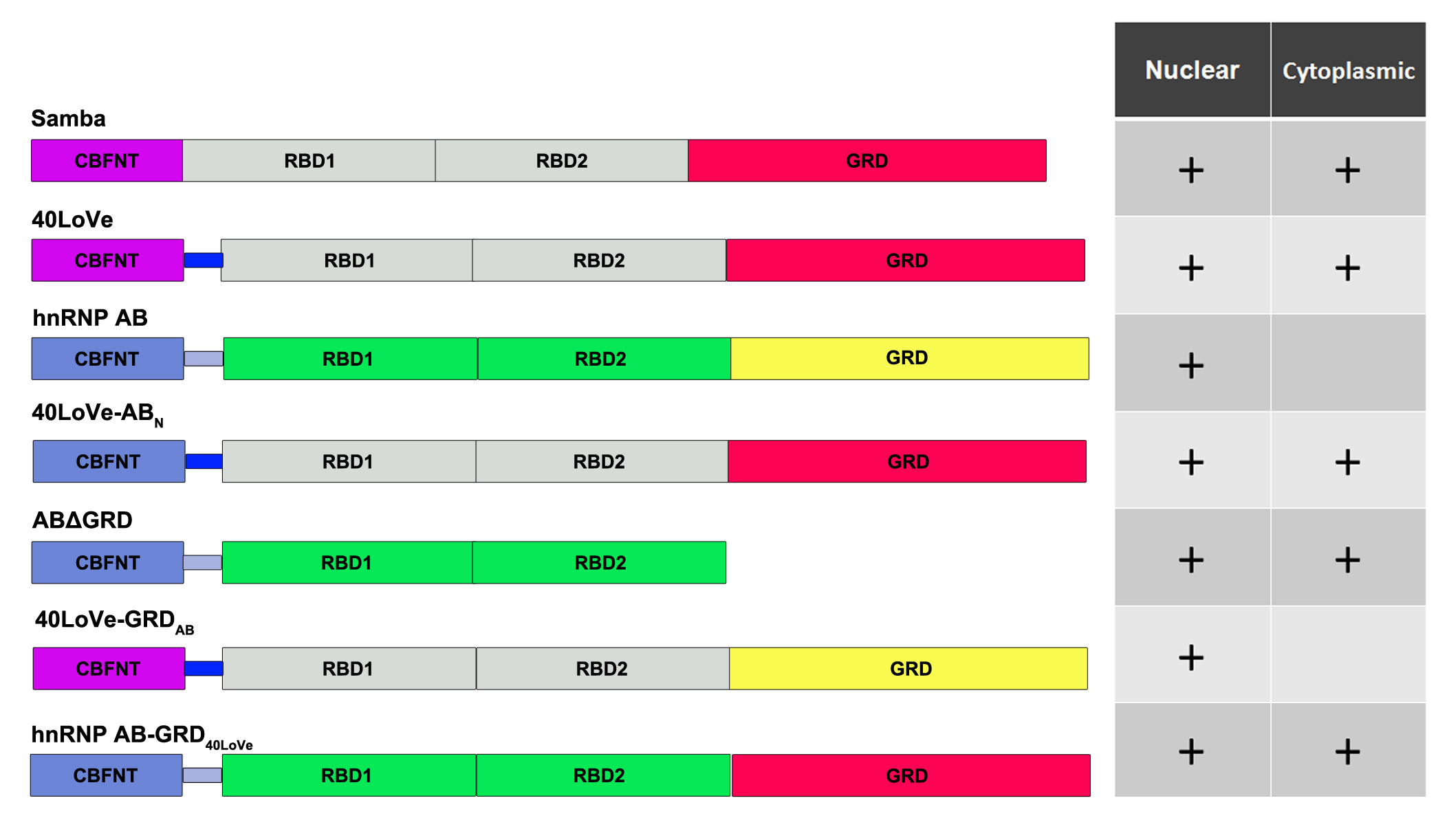

Supplement: Figure S2 — Mutants generated for the determination of the protein domains responsible for the differences in localization between the three proteins. 40LoVe-ABN was constructed using the N-terminus of hnRNP AB form start to nucleotide 270/amino acid 90 and the rest of 40LoVe protein from nucleotide 273/amino acid 91 to the stop codon. ABΔGRD is hnRNP AB with a deleted c-terminus form nucleotide 630/amino acid 210 with an added stop codon. 40LoVeGRDAB was constructed with 40LoVe from start codon to nucleotide 666/amino acid 222 fused with hnRNP AB from nucleotide 633/amino acid 221 to stop codon. hnRNP AB-GRD40LoVe was constructed with hnRNP AB from start codon to nucleotide 630/amino acid 210 fused with 40LoVe from amino acid 690/amino acid 230 to stop codon. All constructs are fused with a FLAG-tag at the C-terminus. (TIF) [file pone.0085026.s002.tif]

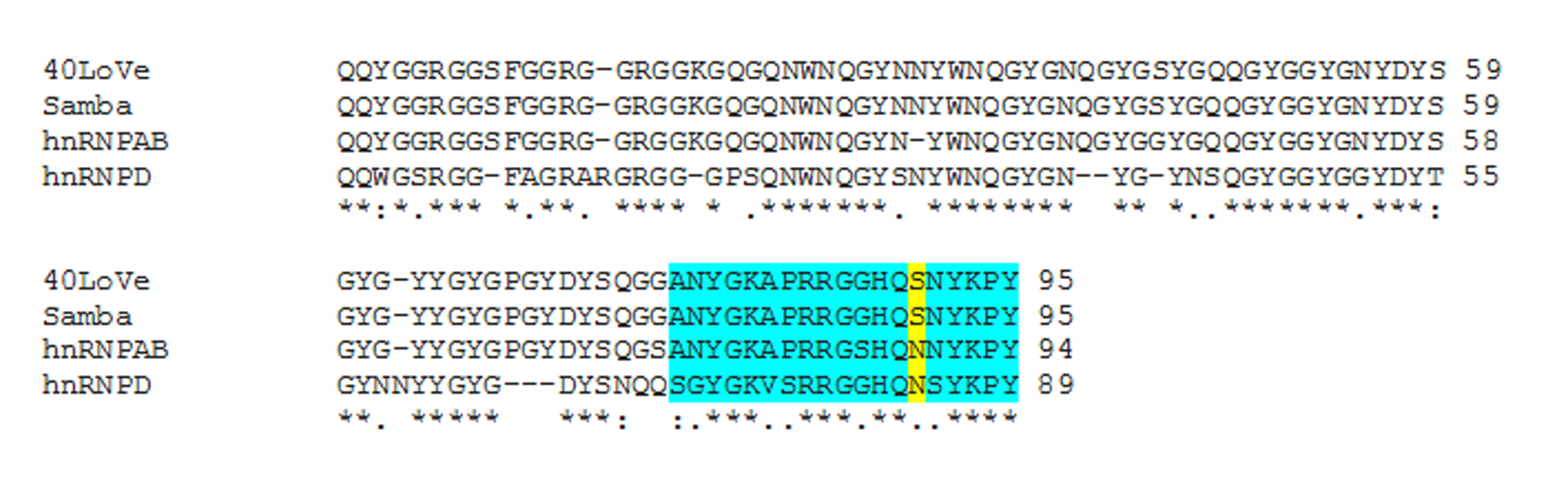

Supplement: Figure S3 — Alignment of the GRD domains of 40LoVe/Samba, hnRNP AB and the human hnRNP D (GI: 51477711). DNS is the 19 amino acid sequence highlighted in turquoise that has been shown to be responsible for shuttling in hnRNP D. Two out of the three differences in the GRD domain between 40LoVe/Samba and hnRNP AB are located in this 19 amino acid sequence. The yellow highlighted amino acid is the one likely responsible for differences in localization between 40LoVe/Samba and hnRNP AB. This amino acid is an Asparagine in hnRNP D and hnRNP AB, which both are strictly nuclear, but it's a Serine in 40LoVe/Samba which show both nuclear and cytosolic localization. (TIF) [file pone.0085026.s003.tif]
