# Supplementary material for: 40LoVe and Samba Are Involved in Xenopus Neural Development and Functionally Distinct from hnRNP AB
Source: PLoS One. 2014 Jan 15;9(1):e85026. doi: 10.1371/journal.pone.0085026 (PMC3893134; doi:10.1371/journal.pone.0085026)
Supplement: Table S1 — Constructs and primers used for each construct generation and the RT-PCRs. (DOCX) [file pone.0085026.s004.docx]

**Table S1**

| **Construct** | **Primer Name** | **Primer Sequence** |
| --- | --- | --- |
| **pCS108-Flag_N_-Samba** | F/EcoRI Flag-SAMBA | AGGAATTCATGGACTACAAGGACGACGATGACAAGAAATCCGACTCCGAGCAGCAGTA |
|  | R/ NotI SAMBA | AGGCGGCCGCTTACCATAGTTTGCACCGCCC |
| **pCS108-5’UTR-hnRNPAB- Flag_C_** | F/EcoRI-5UTR-hnRNPAB | AGGAATTCAATTTGGCGATTGTTGGCGCTTGTGCTTTC |
|  | R/FLAG-NotI-hnRNPAB | CTGCGGCCGCTTATTTGTCATCGTCATCCTTGTAGTCGTATGGCTTGTAGTTATTCTGGTGG |
| **pCS108-5’UTR-40LoVe-Flag_C_** | F/EcoRI5UTR-Samba | AGGAATTCTCGTCGACCCACGCGTCCGCTTGGAGGAATTTGGC |
|  | R/Flag-NotI-Samba | ctgcggccgcttaTTTGTCATCGTCATCCTTGTAGTCcctctgatgtcccccacgtc |
| **pCS108-GFP-SambaΔGRD** | F/EcoRI Flag-SAMBA | AGGAATTCATGGACTACAAGGACGACGATGACAAGAAATCCGACTCCGAGCAGCAGTA |
|  | R/SambaNoGRD | TTTTCTAGAttaaatctttatctcacacttg |
| **pCS108-GFP-CBFNT domain** | F/ClaI-GFP | AGCATCGATATGGGGATCCTGAGTAAAGGAGAA |
|  | R/ClaI-CBFT | TTTATCGATttcaaagtagtctttcaagtccttt |
| **pCS108-R-40LoVe-Flag_C_** | F/Resc-Samba | AGGAATTCATGGGAGGATCGGATTCGGAACAACAATACATGGAAACGAACGCCG |
|  | R/ NotI SAMBA | AGGCGGCCGCTTACCATAGTTTGCACCGCCC |
| **pCS108-R-hnRNPAB –Flag_C_** | F/Resc-hnRNPAB | AGGAATTCATGGGAGGATCGGATACGGAACAACAATGTCTAGAAACGAACGCCGAGAACG |
|  | R/FLAG-NotI-hnRNPAB | CTGCGGCCGCTTATTTGTCATCGTCATCCTTGTAGTCGTATGGCTTGTAGTTATTCTGGTGG |
| **pCS108-GFP-hnRNPAB_N_-40LoVe_C_-Flag_C_** | F/Resc-hnRNPAB | AGGAATTCATGGGAGGATCGGATACGGAACAACAATGTCTAGAAACGAACGCCGAGAACG |
|  | R/hnRNP AB-N(SalI) | aaGTCGACcacctcaccaaactttgcaaagtagtctttcaagtcc |
|  | F/40LoVe-C (SalI) | aaGTCGACtctgactgcacaatcaagatggaccccaatacggg |
|  | R/ NotI SAMBA | AGGCGGCCGCTTACCATAGTTTGCACCGCCC |
| **pCS108-GFP-hnRNPABΔGRD** | F/Resc-hnRNPAB | AGGAATTCATGGGAGGATCGGATACGGAACAACAATGTCTAGAAACGAACGCCGAGAACG |
|  | R/ABΔGRD | tttgcggccgctcactttatctcacacttgcttcc |
| **pCS108-GFP-40LoVe-GRD_AB_-Flag_C_** | F/Resc-Samba | AGGAATTCATGGGAGGATCGGATTCGGAACAACAATACATGGAAACGAACGCCG |
|  | R/40LoVeNoGRD | tttGTCGACctttatctcacacttgcttcc |
|  | F/AB-GRD | aaaGTCGACattgcacaaccaaaagaagtgtatcagc |
|  | R/FLAG-NotI-hnRNPAB | CTGCGGCCGCTTATTTGTCATCGTCATCCTTGTAGTCGTATGGCTTGTAGTTATTCTGGTGG |
| **pCS108-GFP-hnRNP AB-GRD_40LoVe_-Flag_C_** | F/Resc-hnRNPAB | AGGAATTCATGGGAGGATCGGATACGGAACAACAATGTCTAGAAACGAACGCCGAGAACG |
|  | R/40LoVeNoGRD | tttGTCGACctttatctcacacttgcttcc |
|  | F/AB-GRD | aaaGTCGACattgcacaaccaaaagaagtgtatcagc |
|  | F/EcoRI Flag-SAMBA | AGGAATTCATGGACTACAAGGACGACGATGACAAGAAATCCGACTCCGAGCAGCAGTA |
| **RT-Primers**  **40LoVe/Samba** | F/EcoRI-Samba | AGGAATTCATGTCCGACTCCGAGCAGCAG |
|  | R/RT-Samba | TCCCGTATTGGGGTCCATCTTGATTG |
| **RT-Primers**  **Actin** | F-Actin | CCATTGGTAACGAGCGTTT |
|  | R-Actin | GAGGGGCCAGACTCATCATA |
| **RT-Primers**  **Sox2** | F-Sox2 | GAG GAT GGA CAC TTA TGC CCA C |
|  | R-Sox2 | GGA CAT GCT GTA GGT AGG CGA |
| **RT-Primers**  **Chordin** | F-Chordin | CCT CCA ATC CAA GAC TCC AGC AG |
|  | R-Chordin | GGA GGA GGA GGA GCT TTG GGA CAA G |
| **RT-Primers**  **Ntub** | F-Ntub | ATG CTG ATC TAC GCA AAC |
|  | R-Tub | AGA TAG CAG CTA CTG TGA G |
| **RT-Primers**  **Sox10** | F-Sox10 | CAGAGCAACCCTCTACATCTC |
|  | R-Sox10 | GTGTAGTATAGACTGGCTGTTCC |
